# Plastid DNA is a major source of nuclear genome complexity and of RNA genes in the orphan crop moringa

**DOI:** 10.1186/s12870-024-05158-6

**Published:** 2024-05-22

**Authors:** Juan Pablo Marczuk-Rojas, Antonio Salmerón, Alfredo Alcayde, Viktor Isanbaev, Lorenzo Carretero-Paulet

**Affiliations:** 1 Department of Biology and Geology, University of Almería, Ctra. Sacramento s/n, Almería, 04120 Spain; 2 “Pabellón de Historia Natural-Centro de Investigación de Colecciones Científicas de la Universidad de Almería” (PHN-CECOUAL), University of Almería, Ctra. Sacramento s/n, Almería, 04120 Spain; 3 Department of Mathematics and Center for the Development and Transfer of Mathematical Research to Industry (CDTIME), University of Almería, Ctra. Sacramento s/n, Almería, 04120 Spain; 4 Department of Engineering, University of Almería, Ctra. Sacramento s/n, Almería, 04120 Spain

**Keywords:** Organellar genomes, Genome structure and evolution, *isrR* genes, Moringa, NUPTs, Non-coding RNAs, Small RNAs, rRNA genes, tRNA genes

## Abstract

**Background:**

Unlike Transposable Elements (TEs) and gene/genome duplication, the role of the so-called nuclear plastid DNA sequences (NUPTs) in shaping the evolution of genome architecture and function remains poorly studied. We investigate here the functional and evolutionary fate of NUPTs in the orphan crop *Moringa oleifera* (moringa), featured by the highest fraction of plastid DNA found so far in any plant genome, focusing on (i) any potential biases in their distribution in relation to specific nuclear genomic features, (ii) their contribution to the emergence of new genes and gene regions, and (iii) their impact on the expression of target nuclear genes.

**Results:**

In agreement with their potential mutagenic effect, NUPTs are underrepresented among structural genes, although their overall transcription levels and broadness were only lower when involved exonic regions; the occurrence of plastid DNA generally did not result in a broader expression, except among those affected in introns by older NUPTs. In contrast, we found a strong enrichment of NUPTs among specific superfamilies of retrotransposons and several classes of RNA genes, including those participating in the protein biosynthetic machinery (i.e., rRNA and tRNA genes) and a specific class of regulatory RNAs. A significant fraction of NUPT RNA genes was found to be functionally expressed, thus potentially contributing to the nuclear pool.

**Conclusions:**

Our results complete our view of the molecular factors driving the evolution of nuclear genome architecture and function, and support plastid DNA in moringa as a major source of (i) genome complexity and (ii) the nuclear pool of RNA genes.

**Supplementary Information:**

The online version contains supplementary material available at 10.1186/s12870-024-05158-6.

## Background

Transposable elements (TEs) and gene and genome duplications are considered the main molecular forces behind the evolution of plant genome architecture and function [1]. TEs are by far the largest and most variable part of plant genomes [2]. Because of their mobile nature and their propensity to leave traces in their wake across the genome in the form of interspersed repeated sequences, they have traditionally been considered mutagenic and referred to as junk or selfish DNA. However, recent advances in genomics and phenomics are shifting our view of TEs as great contributors to genetic variation on which selection can operate, producing a wide variety of changes in plant gene expression and function with potential adaptive roles on plant evolution [3–6]. New genes and gene structures, in turn, are being continuously added and lost by genomes in a lineage-specific manner. Newly acquired or rearranged genes can evolve novel and/or specialized gene products and/or regulatory functions, ultimately determining to a large degree phenotypic differences between organisms, populations, and species. Several molecular mechanisms are known to be involved in the creation of new genes and gene structures, including exon shuffling and duplication, gene fusion and fission, domestication of TEs, horizontal gene transfer, *de novo* gene origination, and, prominently, gene and genome duplications [7–9]. Of these, gene and genome duplication are considered the main source of raw genetic material upon which mutation and selection, as well as other evolutionary forces, can act upon, ultimately resulting in new and novel gene and gene functions. As a result, the mechanisms that determine their retention in genomes have received much attention [10–14].

However, other genomic sources with potential roles in the origin of evolutionary innovation and adaption are less studied, including the well-known copy of stretches of plastid DNA of different sizes and their subsequent integration into plant nuclear genomes, giving rise to the so-called nuclear plastid DNA sequences (NUPTs) [15]. Although the origins and the evolutionary paths of insertions of organelle DNA into the nuclear genome are probably diverse, they generally involve double-strand breaks and DNA damage and thus are potentially mutagenic [16, 17]. Furthermore, the uncontrolled proliferative insertion of plastid DNA might lead to the unnecessary ‘obesity’ of the nuclear genome. As a result, organellar DNA insertions are generally expected to be neutral or eventually deleterious and selected against [16, 17]. Indeed, most recent plastid DNA insertions are expected to diverge, decay, rearrange, fragment or vanish over evolutionary time, a process that appears to occur rapidly and probably involves mutation, TEs, other DNA sequences unrelated to organelle DNA and replication slippage [16–18].

Plastid genes, in turn, are expected to be inactive upon arrival in the nuclear genome because they often do not encode for complete open reading frames and/or lack the regulatory motifs required for proper gene expression in the nucleus [16, 17]. Furthermore, epigenetic regulation, and prominently DNA methylation and histone tail modification, commonly reported to inhibit the activity of mobile DNA and other types of extraneous DNA, has also been associated with the transcriptional repression of integrated organellar DNA [17, 19–21]. As a consequence, plastid genes typically show low or null expression in the nucleus and will likely evolve as nonfunctional pseudogenes or non-coding sequences [22, 23]. However, as noted in [24–26], expression of NUPT genes in the nucleus do actually occur in plants, so assumptions of nonfunctionality for nuclear genes of plastid origin must be taken with caution. Indeed, on a few occasions, newly arrived organellar genes have been reported, (i) to gain expression capabilities in the nucleus, (ii) to reshape nuclear genes by adding extra coding (exon) or regulatory sequences, or (iii) to proliferate as tandemly arrayed clusters or in distant regions of the nuclear genome [17, 19, 20].

As a result of the usually rapid decay and functional inactivation of NUPTs, in most species organelle DNA represents only a small fraction of less than 0.1% of the nuclear genome, with very few showing more than 1% [17]. However, we recently reported a strong enrichment of NUPTs in the nuclear genome of the orphan crop *Moringa oleifera* Lam. (moringa), representing the largest fraction of plastid DNA so far reported for a plant genome [27]. NUPTs in moringa were found to be formed through two events separated in time, namely I and II, with NUPTs from every event showing markedly distinctive features [28]. While younger NUPTs from episode II showed seemingly random origins throughout the chloroplast genome, a wide range of sizes, preferential location in hotspots, a weak negative correlation between sequence identity and size and, when found in clusters, no collinear arrangement with the plastid genome [28], older NUPTs from episode I were featured by a narrower distribution of sizes, their origin from a few short regions in the chloroplast genome and a preferential collinear arrangement with their plastid ancestors when found in clusters [28].

Therefore, a question that immediately arises is about the molecular and evolutionary forces that may have operated on specific plant lineages promoting the fixation of massive amounts of organellar DNA in the nuclear genome. Here, we explore the functional and evolutionary fate of NUPTs using a chromosome-scale assembly of the moringa genome [29]. We focus our analysis on (i) potential biases in their distribution in relation to different nuclear genomic features, (ii) their contribution to the emergence of new genes and gene regions, and (iii) their impact on the expression of target nuclear genes. Our results support NUPTs as a major source of nuclear genome complexity and of functionally expressed RNA genes and highlight the usefulness of the moringa genome as a model to study the actual impact of NUPTs on the evolution of genome architecture and function.

## Materials & methods

### Reannotation of RNA genes in the moringa nuclear and plastid genomes

Reannotation of RNA genes in the moringa chloroplast genome [30] was performed by scanning the RFAM v14.10 database of non-coding RNA families [31] using the command *cmscan* from Infernal v1.1.4-1 [32]. The tRNA genes found by Infernal were completed by merging with the original annotation reported in [30] and the results obtained through tRNAscan-SE v 2.0.12 [33] using the options -G and -O to search for tRNA from the three domains (eukarya, prokarya and archaea) and organellar genomes, respectively. tRNA genes found in the moringa nuclear and plastid genomes were further classified as nuclear, mitochondrial or plastid according to the annotation of the best hit resulting from BLASTN v2.12.0+ [34] searches of a database of *Arabidopsis thaliana* tRNA genes retrieved from PltRNAdb [35], selecting word size 11 and E-value 10^− 2^ as settings.

### Analysis of the distribution of NUPTs across genomic features

The genome assembly and genomic feature (gff3) files for the moringa nuclear were retrieved from [29]. The genome assembly file for the moringa plastid genome was retrieved from the NCBI Reference Sequence (NCBI-RefSeq) database (https://www.ncbi.nlm.nih.gov/nuccore/NC_041432) [30]. The tabular BLAST file containing the alignments between the moringa nuclear and plastid genomes representing individual NUPTs was retrieved from [28]. Circular plot representations of genomic features were obtained using Circos version 0.69 − 8 [36]. To detect NUPTs overlapping genomic features, the intersect subcommand from BEDTools [37], was employed. The R package *GenomicDistributions* v1.8.0 [38] was used for the base pair overlap count analysis between NUPTs and every other genomic feature. The expected counts of overlapping base pairs between NUPTs and each genomic feature were calculated using the *calcExpectedPartitions* function. Since a large genomic feature in terms of the total number of base pairs it encompasses was expected to overlap more NUPTs by chance than a small one, the *bpProportion* option was activated to account for this bias. Subsequently, *calcExpectedPartitions* performed Pearson’s Chi-squared independence tests with Yates’ continuity correction to assess whether the counts of overlapping base pairs between NUPTs and each genomic feature were significantly different from expected.

### Gene functional annotation and enrichment analysis

Functional annotation terms attached to structural genes found in the moringa genome, including Gene Ontology (GO), Enzyme Commission (EC) and KEGG (Kyoto Encyclopedia of Genes and Genomes) Orthology (KO) groups were retrieved from [29]. Enrichment analysis for detecting over- and under-represented functional terms among NUPT structural genes was performed by means of Fisher’s exact tests [39]. To control for multiple hypotheses testing, the resulting *P* values were corrected according to Bonferroni [40], and those < 0.05 were considered significant. Subcellular localization of nuclear structural genes was predicted by means of DeepLoc 2.0 [41], which generates predictions based on protein language models that only use sequence information.

### Analysis of RNA-seq expression data

Expression values measured in Transcripts Per Million (TPM) for nuclear and plastid genes were obtained from paired RNA-sequencing (RNA-Seq) data from five tissues, i.e., flower, leaf, root, seed and stem, generated in a study of the moringa transcriptome [42], and available at the NCBI Reference Sequence Short Read Sequence Archive (NCBI-SRA) (Table S1). The pool of paired end RNA-Seq reads for each tissue was aligned to the nuclear and plastid genomes simultaneously using the aligner GSNAP v2021-12-2 [43] with one mismatch allowed. The resulting SAM alignment file was sorted by position using the command sort from SAMtools v1.13 [44], and then used to obtain TPM values employing StringTie v2.2.1 [45], a program for transcript assembly and quantitation of RNA-Seq data, on the basis of nuclear and plastid gff3 annotation files. The broadness or tissue-specificity of gene expression was calculated using the Tau index for every gene in the nuclear genome employing the method described in [46] and the expression values from each of the five tissues. The Tau index ranges from 0, indicating broader unspecific expression, to 1, reflecting narrower specific expression [47]. Significance in the departure of the fraction of expressed versus unexpressed genes from that expected by chance for specific classes of genes was assessed through Fisher’s exact tests [39]. The significance of the differences in overall expression, measured by TPM values, or in expression broadness, measured by Tau indexes, between subsets of genes, was assessed through Wilcoxon´s rank tests [48].

For the GSNAP alignments, a Single Nucleotide Polymorphism (SNP) file containing editing sites predicted in the plastid transcripts was used to distinguish them from nuclear transcripts, and thus ensure that read mapping results reflected actual transcription of NUPT genes. Prediction of editing sites was made by REDItools v2.0 [49], a collection of python scripts for the prediction RNA editing sites, from a sorted BAM file containing alignments between the pool of reads from the five tissues considered together and the plastid genome. The alignments were performed though GSNAP v2021-12-27 [43], which generated a SAM alignment file that was later converted to bam and sorted by position using the commands view and sort from SAMtools v1.13 [44], respectively.

## Results

### Biased distribution of NUPTs across moringa nuclear genomic features

We used here our previous classification of NUPTs identified in the moringa genome based on the posterior probabilities resulting from assigning a NUPT to the two main peaks detected when fitting Gaussian mixture models to their distribution of relative ages measured in terms of percentage sequence identity [28]. Each of these two peaks grouped NUPTs as formed in two distinct episodes or events separate in time: older NUPTs from class I, with 776 occurrences, and younger NUPTs from class II, including 3,855 representatives. 572 additional NUPTs could not be confidently assigned to any class using a threshold posterior probability of 95% and were considered as a separate class named ‘unclassified’, perhaps representing an intermediate evolutionary process in the fate of NUPTs in nuclear genomes (Table S2) [28]. To examine the spatial distribution of the NUPTs across the different sequence features found in the moringa nuclear genome, we employed the structural annotation of a chromosome-scale assembly, i.e., AOCCv2 [29] which was completed by further classifying tRNA genes as plastid, mitochondrial or nuclear. 95.76% of the genome could be categorized into 12 genomic features, including structural genes, Transposable Elements (TEs), other repeats, NUPTs plus nine different categories of RNA genes, while the rest was deemed as “other DNA” (Table S2). In addition, TEs were further reclassified in two classes, i.e., class I (retrotransposons) and class II (DNA transposons), six orders and 18 superfamilies (Table S3) following the classification system reported in [50], which was selected to unify the two nomenclatures used in the original annotation [29].

A total of 4,885 genomic features were found to be affected by 4,754 out of 5,302 NUPTs (Table 1), which we will refer to as NUPT genomic features. The majority of the NUPT genomic features corresponded to TEs (2,317) and plastid tRNA genes (912) (Table 1 and Table S4). 3,716 NUPTs were found in two or more specific genomic features (Table 1). The percentage of genomic features affected by NUPTs varied widely, ranging between 0%, for spliceosomal and other RNA genes and more than 99% for plastid tRNA genes (Table 1). Aside from nuclear plastid tDNA, two additional categories of RNA genes for which the majority of members were affected by NUPTs were self-splicing intron RNA genes (96.06%) and prokaryotic rRNA genes (93.21%) (Table 1). Within every category of NUPT genomic features, the percentage of them aligning in their entirety with plastid DNA was also highly variable, ranging between 5.61% for structural genes to more than 99%, as was the case for mitochondrial and plastid tRNA, as well as self-splicing intron RNA genes (Table 1).

**Table 1:** Summary of NUPT genomic features

NUPT genomic feature	Total number	Total percentage (%) in the genome	Percentage (%) fully covered by plastid DNA	Number of NUPTs^2^
Structural gene^1^	428	1.88	5.61	657
TE	2,317	1.81	40.40	3,858
Other repeats	80	0.10	90	105
Eukaryotic rRNA gene	132	5.19	27.27	264
Prokaryotic rRNA gene	302	93.21	55.96	832
Nuclear tRNA gene	112	18.33	95.54	119
Mitochondrial tRNA gene	48	81.36	100	48
Plastid tRNA gene	912	99.89	99.56	885
Self-splicing intron RNA gene^3^	512	96.06	98.63	633
Regulatory RNA genes^4^	42	11.2	69.05	62
Spliceosomal RNA gene	0	0	0	0
Other RNA genes	0	0	0	0
Two or more genomic features^2^	4,416	-	-	3,716

^1^Including the 1 Kb regions upstream and downstream of the ATG and stop codons, respectively

^2^Note that there are NUPTs overlapping two or more genomic features and so the total number is higher than the actual number of NUPTs.

^3^Group I and group II introns

^4^Only iron stress-repressed RNA (*isrR*) genes

**Fig. 1:**
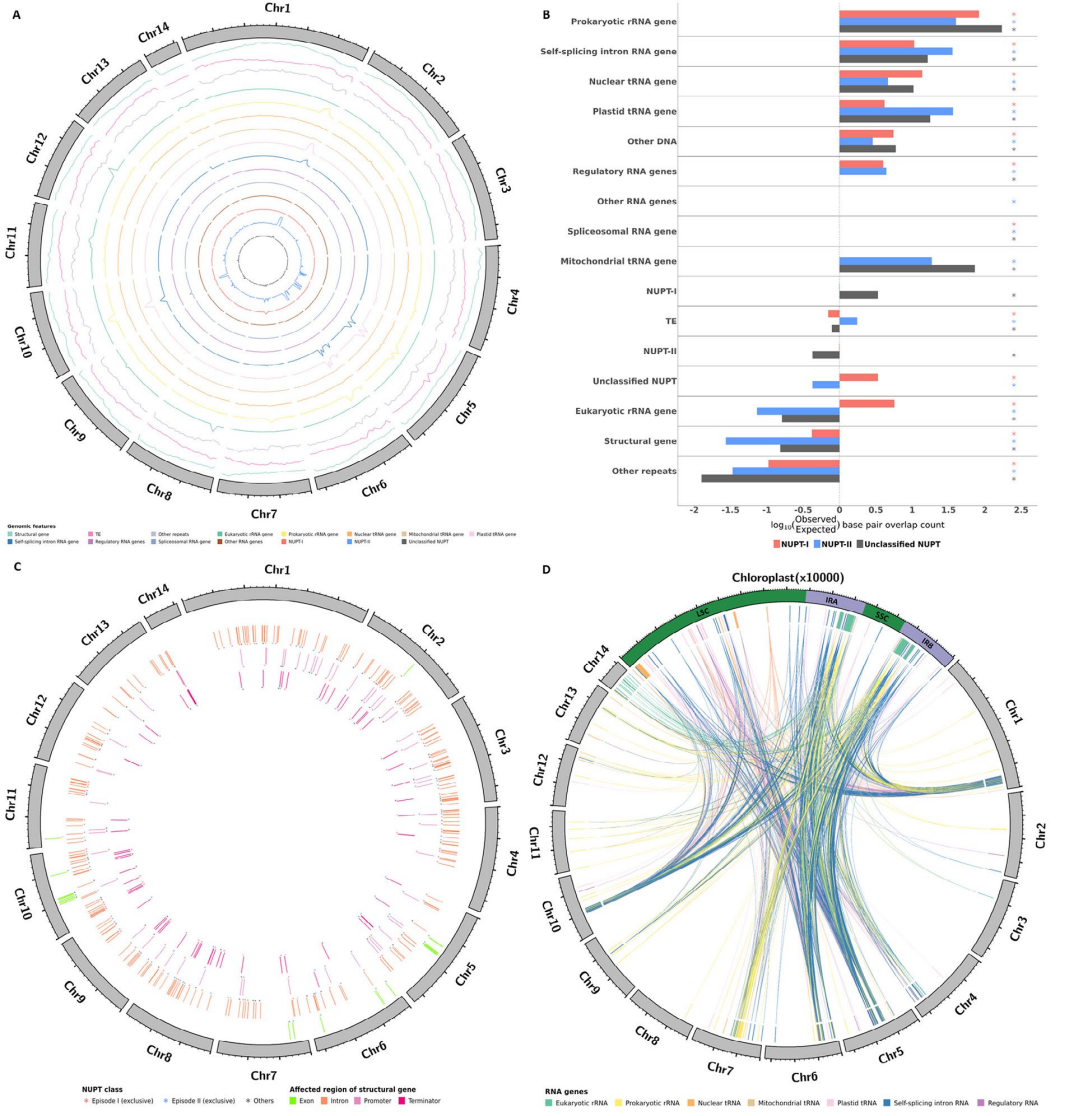
Distribution of NUPTs across moringa nuclear genomic features. (**A**) Circos plot representation of genomic features detected in the moringa nuclear genome. The 14 nuclear chromosomes are represented as grey filled blocks forming a circle and arranged clockwise. Nuclear chromosomes are drawn to scale, with lengths proportional to size and expressed in Mb. Each inner ring contains a line plot representing the density distribution of specific genomic features in windows of 500,000 bp. Structural genes include the 1 Kb region upstream of the ATG codons, exons, introns and the 1 Kb region downstream of the stop codon. (**B**) Overlap count analysis between NUPTs partitioned by class and every other genomic feature. For easier visualization, overlap values are displayed as log10-transformed observed/expected base pair overlap counts. Colored asterisks indicate the counts of observed overlapping base pairs between NUPTs and every other genomic feature were significantly higher or lower than expected, respectively, as resulting from Pearson’s Chi-squared tests with Yates’ continuity correction. (**C**) Circos plot representation of NUPT structural genes detected in the moringa nuclear genome. The nuclear genome is depicted as in panel **A**. NUPT structural genes are shown as tiles colored according to the gene region affected. Colored asterisks indicate the class of the NUPT(s) present in the structural genes affected: I, II and other, i.e., one episode and the another and / or unclassified, respectively. (**D**) Circos plot representation of NUPT RNA and plastid RNA genes detected in the moringa nuclear and plastid genomes, respectively. The nuclear genome is depicted as in panel **A**. The chloroplast chromosome is represented as a green filled block located at 12 o’clock and has been upscaled to occupy a quarter of the image circumference; its size unit was set to 10,000 bp. NUPT RNA and plastid RNA genes are shown as tiles colored according to their category. NUPT RNA genes and their donor gene regions in the chloroplast genome are connected through links whose colors indicate whether they were annotated to the same category or not, in which case they are shown in grey. LSC, Large Single Copy; IRA, Inverted Repeat A; IRB, Inverted Repeat B; SSC, Small Single Copy

Next, we examined any potential spatial biases in the distribution of NUPTs with respect to specific genomic features, i.e., whether specific genomic features were more or less tolerant to host NUPTs. First, visually, by graphically representing the arrangement of NUPTs from every class plus every other feature found in the moringa genome along the 14 chromosomes using Circos plots (Fig. 1A). The frequency of genomic features was represented in each case as density plots in windows 500 kb in length. We then searched for any putative overlaps in density peaks between genomic regions corresponding to NUPTs partitioned by class and the rest of genomic features. As expected, older NUPTs from episode I, which were found to be apparently distributed homogenously across chromosomes [28], do not show any apparent peaks in the distribution (Fig. 1A). A similar pattern could also be observed for unclassified NUPTs (Fig. 1A). In turn, younger NUPTs from episode II, which were found to be concentrated in hotspots [28], collocated with peaks in the density distribution of different classes of RNA genes, including self-splicing intron, plastid tRNA and prokaryotic rRNA (Fig. 1A).

Next, we tried to statistically substantiate putative biases in the distribution of NUPTs partitioned by class against every specific genomic feature by comparing observed versus expected base pair overlap counts (Fig. 1B and Table S5). In the context of this study, an overlap between NUPTs and a specific genomic feature significantly greater than expected, i.e., an enrichment in NUPTs across that specific feature would indicate higher tolerance to this type of insertion, i.e., selection being less efficient to remove NUPTs associated to that specific genomic feature. In turn, impoverishment in overlaps between NUPTs and a specific genomic feature may be interpreted as insertions of NUPTs in these being potentially deleterious and consequently selected against. Based on the results from performing Pearson’s Chi-squared independence tests with Yates’ continuity correction, prokaryotic rRNA genes, tRNA genes, self-splicing intron RNA genes, regulatory RNA genes, and other DNA were highly enriched for NUPTs from one and another episode whereas structural genes, other repeats, spliceosomal RNA genes and other RNA genes were highly impoverished (Fig. 1B and Table S5). Features such as eukaryotic rRNA genes, TEs and unclassified NUPTs, showed opposite trends of enrichment depending on the NUPTs´ formation event (Fig. 1B and Table S5).

### NUPTs are preferentially retained among specific superfamilies of class I retrotransposons

We further explored biases in the association between TEs and NUPTs. In order to address the differential impact of TEs in NUPTs reshuffling, amplification, removal, and fragmentation over time, TEs were previously classified by class, order and superfamily (Table S5). The majority of NUPTs’ TEs belong to class I, more specifically corresponding to retrotransposons from superfamilies LINE/L1, LINE/I, LTR/Gypsy and SINE/tRNA (Table S6). We next searched for any putative overlaps between every class of NUPTs and TEs partitioned by class and order by visually representing their density peaks along the 14 chromosomes of the moringa genome using Circos plots (Fig. S1). Finally, we compared observed versus expected base pair overlap counts between NUPTs from every class and TEs partitioned by class, order, and superfamily to statistically substantiate putative biases in the distribution of NUPTs (Table S7 and Fig. S2-4). In contrast to the previous results from overlap analysis between NUPT from each class and all TEs considered together (Fig. 1B), we found a consistent enrichment of NUPTs from every class and class I TEs, more specifically, retrotransposons belonging to superfamilies LINE/L1, LINE/I, LTR/Gypsy and SINE/tRNA, while a consistent impoverishment could be observed with respect to class II DNA transposons (Table S7 and Fig. S2-4). In summary, our results supported the preferential association between NUPTs and specific superfamilies of retrotransposons.

**Fig. 2:**
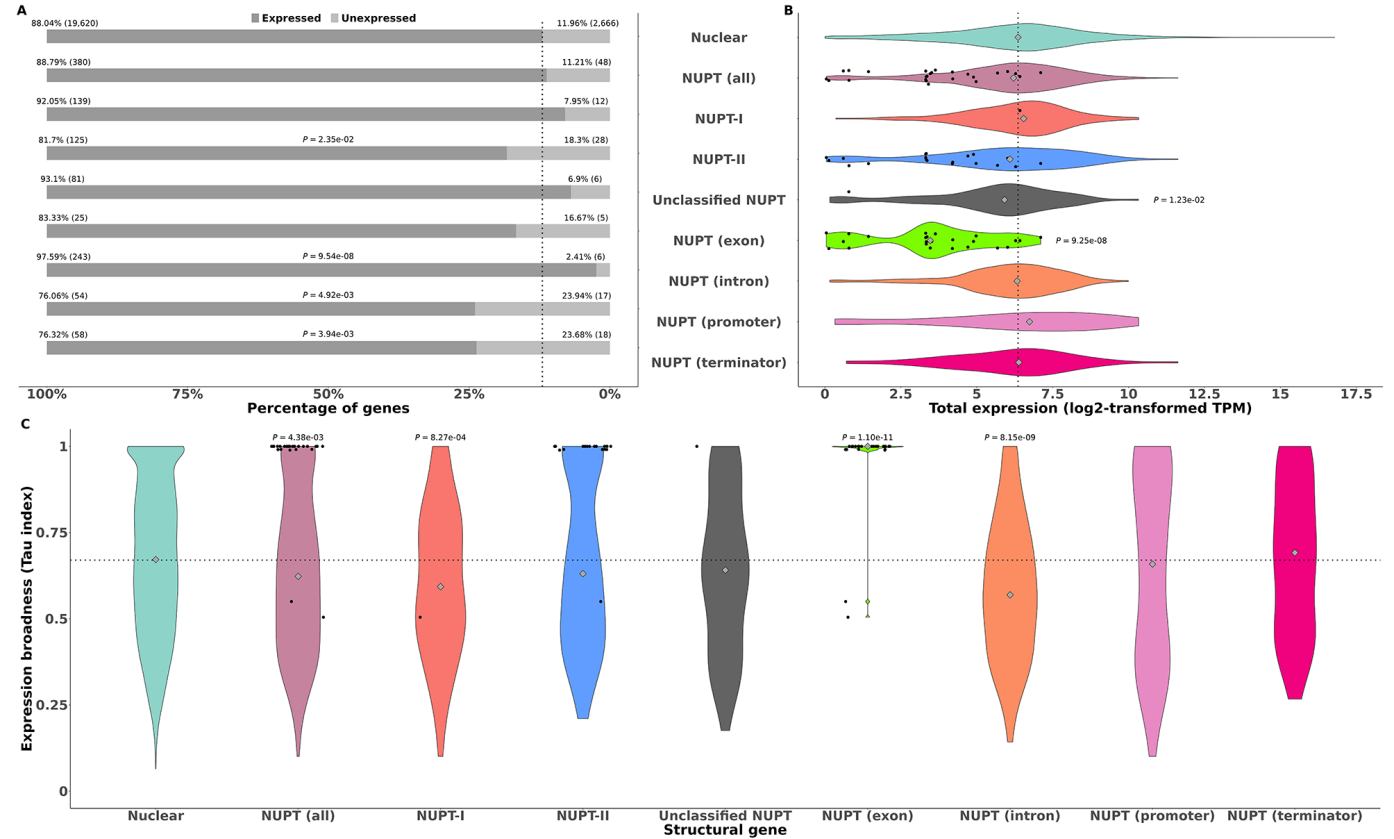
Expression levels and broadness of NUPT structural genes partitioned by class and gene region affected plus non-NUPT ones. (**A**) stacked bar plot representing the percentage of expressed versus unexpressed genes. The dotted line indicates the total percentage of unexpressed structural genes. Significant *P*-values from performing Fisher’s exact tests for each category of NUPT structural genes versus the whole set of structural genes are shown. (**B**) Violin plot representing log2-transformed expression values measured in Transcripts Per Million. Only expressed structural genes were considered. (**C**) Violin plot representing expression broadness measured by means of Tau index for every gene in the category. In all cases, individual NUPT genes affected in exonic regions are depicted as black dots. Grey diamonds inside violin plots in (**B**) and (**C**) represent median expression level and broadness, respectively, for each category of structural genes. Dotted lines in plots (**B**) and (**C**) show the median expression level and broadness, respectively, of non-NUPT structural genes. Significant *P*-values in plots (**B**) and (**C**) resulting from performing Wilcoxon’s rank tests between each category of NUPT structural genes and non-NUPT ones are shown

**Fig. 3:**
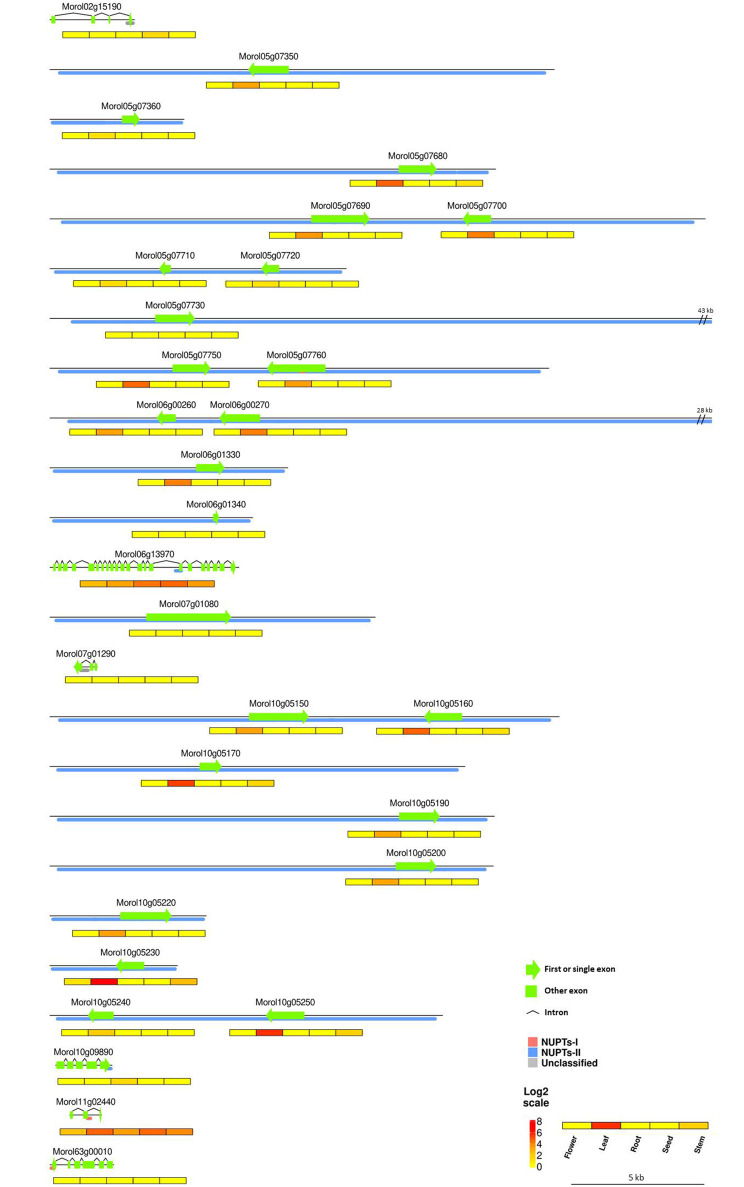
Schematic diagram of the 30 NUPT structural genes affected in exons. Gene models (i.e., exons and introns) are represented. NUPTs are depicted as blocks colored according to their class. Below each NUPT structural gene there is a heat map representation of their expression patterns in five tissues. The colors of the heat map represent log2-transformed expression values measured in Transcripts Per Million. The elements in the diagram are drawn to scale. Some elements were trimmed to adjust the total size of the image; their actual sizes are indicated

**Fig. 4:**
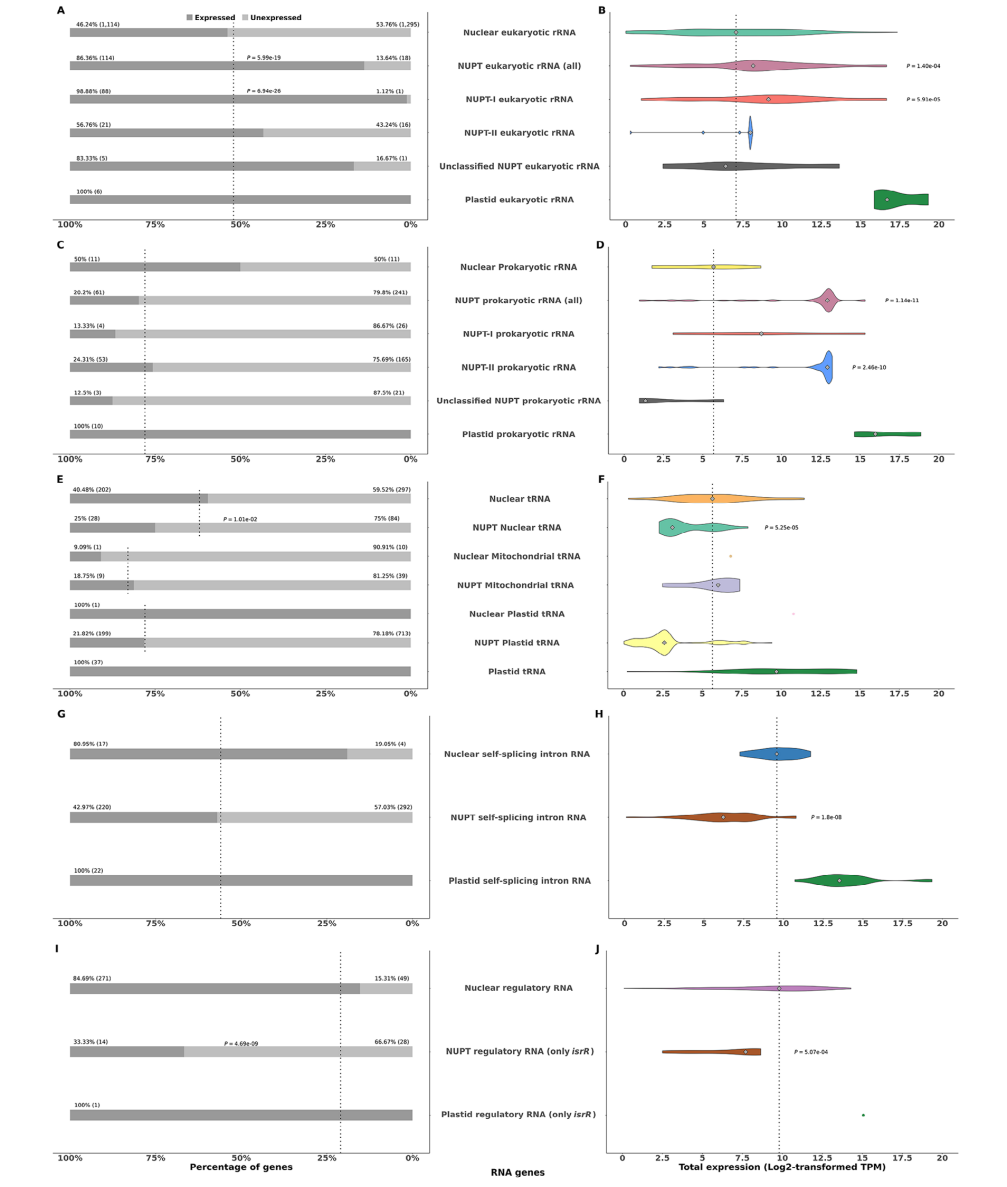
Expression levels of specific classes of NUPT, non-NUPT, and plastid RNA genes. (**A**), (**B**) Eukaryotic rRNA. (**C**), (**D**) Prokaryotic rRNA. (**E**), (**F**), tRNA. (**G**), (**H**) Self-splicing intron RNA. (**I**), (**J**) Regulatory RNA. On the left panels, stacked bar plots representing the percentage of expressed versus unexpressed genes. Dotted lines indicate the total percentage of unexpressed nuclear RNA genes of each class. Significant *P*-values resulting from performing Fisher’s exact tests for each class of NUPT RNA genes versus the whole set of nuclear RNA genes of that class are shown. On the right panels, violin plots representing log2-transformed expression values measured in Transcripts Per Million. Only expressed genes were considered. Groups with fewer than two expressed genes are depicted as colored single dots. Grey diamonds inside violin plots represent median expression level for each class of RNA genes. Dotted lines indicate the median expression of nuclear RNA genes for each category. The dotted lines also indicated the median expression of mitochondrial or plastid tRNA genes. Significant *P*-values resulting from performing Wilcoxon’s rank tests between NUPT and nuclear RNA genes of each class are shown

### NUPTs show differential patterns of retention between structural and RNA genes

As stated previously, structural genes were found to be affected by NUPTs from each class less than expected by chance (Fig. 1B and Table S5), likely reflecting the potential deleterious effects resulting from the insertion of exogenous DNA, especially when affecting coding sequences. However, impoverishment in NUPTs across structural genes could be observed regardless of the region of the gene affected (Fig. S5 and Table S8).

A total of 428 structural genes were affected by 657 NUPTs (Table 1 and S9), including 249 affected in intron regions, 71 in the 1 Kb region upstream of the ATG start codon, considered as the promoter region, 76 inserted in the 1 Kb region downstream of the stop codon, considered the terminator region, and only 30 affected in exons, including 12 single exon genes originating from six individual NUPTs from episode II (Table S10). Although many NUPT structural genes showed one single NUPT (222), the remaining displayed a variable number ranging from two to up to 10 NUPTs (one single gene, *Morol14g00250*) (Table S10). Additionally, two NUPT structural genes (*Morol07g01780* and *Morol05g07670*) were affected by more than one NUPT found in both the promoter and terminator regions.

We explored the spatial arrangement of NUPT structural genes across the moringa nuclear genome. For this purpose, NUPT structural genes from each class, categorized according to the gene regions affected, were graphically represented across the moringa chromosomes in the form of Circos plots (Fig. 1C). 151 structural genes were exclusively affected by NUPTs-I, while 153 and 87 were exclusively affected by NUPTs-II and unclassified ones, respectively; 37 structural genes contain NUPTs from more than one class (Fig. 1C). In general, NUPT structural genes appear to be scattered across all 14 chromosomes and did not show any apparent arrangement in clusters, the only exception being NUPT structural genes affected in exon sequences (Fig. 1C).

In contrast to the underrepresentation of NUPTs among structural genes, the opposite trend could be observed for most categories of RNA genes. This observation was especially significant for organellar tRNA, prokaryotic rRNA and self-splicing intron RNA genes present in the nuclear genome, the majority of which were of plastid origin (Table 1). We further mapped NUPT RNA genes to the region of origin in the plastid genome using a newly obtained annotation of RNA genes in the moringa plastid genome. Our new annotation of the plastid genome detected additional RNA genes, including five eukaryotic rRNA, one regulatory RNA (corresponding to iron stress-repressed RNA genes, *isrR*) plus 22 self-splicing introns (Fig. 1D). Furthermore, in addition to the 36 tRNA genes found in the original annotation, an additional selenocysteine tRNA gene was detected. tRNA genes found in the plastid genome were further classified as plastid, mitochondrial, and nuclear (Fig. 1D). In general, NUPT RNA genes matched RNA genes in the plastid genome that belong to the same category. For example, of the 913 and 59 genes in the moringa nuclear genome annotated as plastid and mitochondrial tRNA, 912 and 48, respectively, corresponded to tRNA genes identically annotated in the plastid genome (Fig. 1D). Of the 611 nuclear tRNA genes found in the nuclear genome, 112 were of plastid origin, 85 out of which were similarly annotated in the plastid genome, while the rest were annotated as plastid tRNA genes (Fig. 1D). A similar situation was observed in the 324 genes annotated in the nuclear genome as encoding for prokaryotic rRNA, 302 of which originated from prokaryotic rRNA genes found in the plastid genome. Regarding the 2,541 eukaryotic rRNA genes found in the nuclear genome, only 132 were derived from the plastid genome, where they were identically annotated, except 36 genes encoding for 5 S rRNA, which corresponded to two 5 S rRNA genes annotated as prokaryotic in the plastid. Another category of RNA genes enriched for NUPTs was that of regulatory RNA genes, 42 out of 375 arising from a single gene found in the plastid annotated as *isrR*. Furthermore, every single of the 512 out of 533 genes annotated as NUPT self-splicing intron in the nucleus proceeded from a gene region identically annotated in the plastid. Finally, the representation of NUPT RNA genes across the 14 chromosomes of the moringa nuclear genome revealed their preferential arrangement in clusters, in contrast to that observed for structural genes (Fig. 1D).

### Functional and expression characterization of NUPT structural genes

We examined whether the presence of NUPTs in structural genes could determine differences in their expression, qualitatively or quantitatively, with respect to the rest of the genes in the genome, using RNA-seq data from five tissues, i.e., flower, leaf, root, seed, and stem [42]. Out of the 428 NUPT structural genes, 380 showed significant expression in at least one of the five tissues, a fraction not significantly different from that found among all structural genes according to Fisher’s exact test (Fig. 2A). When partitioned by class, NUPT-II structural genes featured a fraction of expressed genes significantly smaller than expected by chance, while the fraction of expressed genes among unclassified and NUPT-I ones was higher, although not significantly (Fig. 2A). Furthermore, the fraction of expressed versus unexpressed NUPT structural genes showed deviations from non-NUPT ones depending on the region affected by NUPTs (Fig. 2A). While this fraction was significantly greater in the case of NUPT structural genes affected in introns, the opposite situation was observed for structural genes with NUPTs located in promoter or terminator regions, with no significant differences among those genes affected by NUPTs in exons (Fig. 2A).

Moreover, the overall expression of NUPT structural genes was in general not different from that of non-NUPT ones, when their median values where compared according to a Wilcoxon rank test, either when considered together, partitioned by class or by the gene region affected, except among those affected by unclassified NUPTs and in exons, which featured an overall expression significantly lower (Fig. 2B).

We next checked for differences in expression broadness across five tissues between NUPT structural genes and non-NUPT ones by using the Tau index, i.e., whether the presence of NUPTs resulted in a broader or in a more restricted expression pattern, using the RNA-seq data from each of the five tissues sampled. The values of the Tau index range from 0, indicating wider unspecific expression, to 1, reflecting narrower specific expression [47]. NUPT structural genes showed a significantly broader expression across the five tissues with respect to the rest of structural genes in the genome according to a Wilcoxon’s rank test (Fig. 2C), an effect specifically related to structural genes affected by NUPTs-I in intronic regions (Fig. 2C). In contrast, NUPT structural genes affected in exonic regions showed a significantly narrower expression across the five tissues compared to the rest of structural genes in the genome (Fig. 2C).

Next, we tried to get insights about the potential involvement of the 428 NUPT structural genes in specific biological functions. For this purpose, we first used GO terms. Seven GO functional terms related to chloroplast and photosynthesis molecular functions, biological processes or subcellular locations were found to be significantly overrepresented (Table S11). Similar enrichment tests were also performed using EC numbers, representing a hierarchical classification of chemical reactions catalyzed by enzymes, and KEGG KO terms, describing molecular functions represented in terms of functional orthologs. One EC term (EC:1, grouping oxidoreductases), was found to be significantly overrepresented, whereas two other EC terms (EC:3.5.1.98, histone deacetylase; EC:1.10.3.9, photosystem II oxidoreductase) were found to be marginally overrepresented (Table S12). A KO term (K02704), describing a chlorophyll apoprotein, was found to be enriched among NUPT structural genes, while two other KO terms that also describe chlorophyll apoproteins (K02690 and K02705) were found to be marginally enriched (Table S13). The enrichment found among NUPT structural genes for biological functions and enzymatic activities related to chloroplast functions is not surprising considering that most of the genes annotated with such terms were entirely of plastid origin (Tables S11-14). Indeed, DeepLoc 2.0 predictions of subcellular localization [41], did not find a significantly different number of NUPT structural genes to be imported to the chloroplast (45) than for non-NUPT structural genes (1,982), according to a Fisher’s exact test (*P* = 0.23), as expected if NUPT structural genes had preferentially evolved chloroplast-related functions.

We further examined in detail the 30 NUPT structural genes that were found to be affected in exons. A schematic representation of the corresponding gene models showing the regions covered by NUPTs, their class, and the expression patterns in five representative tissues is shown in (Fig. 3). Most of the 24 single exon genes completely composed of plastid DNA showed either low expression restricted to leaves or null expression and were annotated as encoding for photosystem or other chloroplast-related functions (Table S14). The remaining six NUPT-structural genes were not fully covered by plastid DNA and corresponded to nuclear genes that would have incorporated additional coding regions of plastid origin, i.e., showed signatures of exonization and might therefore encode for novel protein functional domains and, ultimately, biological functions. Four of them showed expression and, therefore, could be functional. For example, *Morol06g13970*, affected by a single NUPT-II spanning the end of intron 15 and the beginning of exon 16, showed the highest expression in all five tissues. *Morol06g13970* was annotated as encoding for the nuclear TPR3-like protein (Table S14), featured by a number of WD40 repeated motifs rich in Asp and Trp residues. WD40 motifs are found in a diverse range of proteins covering a wide variety of plant developmental-related functions ranging from signal transduction and transcription regulation to cell cycle control and apoptosis [51]. A second gene, *Morol10g09890*, annotated as encoding for the cytochrome P450 CYP72A219-like protein, was affected by a NUPT-II that spans the end of its last exon and the beginning of its terminator region and was only marginally expressed in roots (Fig. 3 and Table S14). *Morol11g02440*, highly expressed in all five tissues and affected by a single NUPT-I spanning the end of exon two and the beginning of intron two (Fig. 3), and *Morol02g15190*, only found to be marginally expressed in seeds and affected by an unclassified NUPT that spans its last exon (Fig. 3), had no annotated function (Table S14). BLASTP searches for putative homologous proteins in the Uniprot database v2023_04 [52] yielded translation initiation factor IF-1, an essential component for the initiation of protein synthesis, as the best hit for *Morol11g02440*. In turn, the best retrieved BLAST hit for *Morol02g15190* was annotated as a polyprenol reductase, a key player in the early steps of protein N-linked glycosylation.

### Many NUPT RNA genes are functionally expressed

In a first attempt to assess whether nuclear RNA genes of plastid origin were functional, we examined their expression using our RNA-seq dataset from five tissues. As for the structural genes above, the number and percentage of expressed genes with respect to the total plus the distribution of their expression levels were represented as stacked bar plots and violin plots, respectively, for every class of RNA genes found as enriched for NUPTs (Fig. 4). In every case, the expression of homologous RNA genes present in the plastid genome was also shown for comparison. The fraction of expressed NUPT eukaryotic rRNA genes was found to be significantly higher than expected according to Fisher’s exact test, an effect specifically related to NUPTs-I, while no significant differences were found between NUPT prokaryotic rRNA genes (Fig. 4A, C). The overall expression of NUPT rRNA genes was in general greater than that of non-NUPT ones, with differences being significant for eukaryotic rRNA genes in the case of NUPTs-I and for prokaryotic ones in the case of NUPTs-II (Fig. 4B, D). For the rest of the RNA genes in the nuclear genome, the fraction of genes expressed among those of plastid origin was generally lower than expected for most categories (Fig. 4E, G, I), and, unlike what was observed among rRNA genes, their overall expression was in all cases smaller among those of plastid origin, with significant differences in all cases where a Wilcoxon’s rank tests could be implemented (Fig. 4F, H, J).

## Discussion

The rich fraction of plastid DNA found in the moringa genome provides an unprecedented opportunity to study the impact of NUPTs on the evolution of the architecture and function of nuclear genomes. The results presented here reveal the biased distribution of NUPTs across different genomic features, likely indicating that some genomic regions tolerate the insertion of plastid DNA better than others, i.e., selection being less efficient to remove NUPTs depending on the genomic region affected. For example, NUPTs had been found to be associated with TEs in certain species, in coherence with the role attributed to TEs and other nuclear DNA sequences unrelated to organelle DNA in promoting erosion and rearrangement in the nucleus of recently inserted plastid DNA, although the precise molecular mechanisms involved have not yet been fully elucidated [18, 53]. In moringa, association between NUPTs and TEs has been found to be dependent on (i) the time and mode of origin, with younger NUPTs from episode II found to overlap with TEs significantly more than expected by chance, while the rest of NUPTs being underrepresented among TEs; and (ii) the specific TE superfamily, with NUPTs consistently enriched among retrotransposons belonging to superfamilies LINE/L1, LINE/I, LTR/Gypsy and SINE/tRNA [18, 54]. It remains to be determined whether this preferential association reflects the actual role of specific TEs superfamilies in the evolutionary and functional outcome of NUPTs over time. This association might not necessarily result in the erosion of NUPTs, as revealed the weak, although significant, negative correlation found between size and sequence identity of younger NUPTs-II [28], but rather be suggestive of a role for retrotransposons in NUPT proliferation and increase in copy number after insertion in the nuclear genome, as previously suggested [55, 56].

Not surprisingly, structural genes were consistently found to be hit by NUPTs less than expected by chance, reflecting their likely deleterious effect, especially when integrated in exon coding regions. Of the 30 moringa NUPT structural genes affected in exons, plastid DNA contributed partially to coding sequences only in six of them, while the rest corresponded to single exon-genes made entirely of plastid DNA. Therefore, although the repeated transfer of copies of plastid DNA stretches to the nuclear genome might provide the plant with a source of genetic material to modify preexisting gene functions and/or acquire novel ones, other molecular mechanisms rather than exonization seem to have operated on the moringa lineage promoting the repeated fixation of massive amounts of plastid DNA in the nucleus. However, it should be noted that most NUPTs are expected to diverge in sequence through evolutionary time, resulting in the amelioration of the plastid DNA sequence to the nucleotide composition of its host chromosome, becoming gradually difficult to detect through direct searches of significant identity with the donor plastid genome regions [53]. Thus, NUPTs might still have contributed to ancient functional exon acquisitions more than anticipated [20].

It had also been reported a role for NUPTs in the dissemination of regulatory elements in the promoter or enhancer of specific genes, resulting in a more efficient transcription [57–59]. Although this might be the case for individual NUPTs in moringa, it does not seem to be a general pattern; overall transcription levels of NUPT structural genes were in general not found to be different than that of their non-NUPTs counterparts, with the exception of those affected by unclassified NUPTs, which featured lower expression, those affected at exonic regions, whose expression was found to be lower and more specific, and those affected by NUPTs-I at intronic regions, which displayed a broader expression. Nevertheless, the consistent expression found here, both quantitatively or qualitatively, for NUPT structural and RNA genes taken collectively, suggest (i) they are not preferential targets for transcription repression through hypermethylation or other epigenetic silencing mechanisms as had been previously claimed from studies in other species [17, 19–21], or (ii) transcription repression only affect specific subclasses of NUPT genes, such as exon structural ones.

In contrast to structural genes, most categories of RNA genes considered in our study were consistently found to contain plastid DNA more than expected by chance. Upon arrival to the nuclear genome, and similarly to structural genes, RNA genes are not expected to be functional. However, we found here a significant fraction of NUPT RNA genes from different categories showing functional expression, in some cases at higher levels than the corresponding non-NUPT counterparts. This was the case for nuclear genes of plastid origin annotated as eukaryotic or prokaryotic rRNA, although it remains to be determined whether NUPT rDNA can contribute to the cytosolic pool of rRNA and ribosomes. The hundreds or thousands of rRNA genes commonly found in eukaryotic nuclear genomes are remarkably well conserved in sequence, with gene conversion and/or concerted evolution through unequal crossover being the major driving force underlying sequence conservation by sweeping away any newly acquired mutations [60]. This provides a suggestive mechanism to explain the amelioration of prokaryotic rDNA sequences of plastid origin to the nuclear genome. Additionally, extraribosomal functions have been suggested for repetitive tandems of rDNA, including the following: (i) evolve as rRNA-derived RNA fragments (rRFs), a novel class of regulatory small noncoding RNAs (sncRNA), whose exact functions have not been elucidated yet [61–63]; (ii) contribute to the maintenance of genome stability, being particularly sensitive to genomic stresses and acting as a source of adaptive response [64].

NUPTs also constitute a major source of tRNA genes in the nuclear genome. The occurrence of organellar tRNA genes had previously been observed in the nuclear genomes of different plant species [65–67]. However, NUPT tDNA commonly represents a minor, although highly variable, fraction of the total tDNA content, while in the case of moringa, 67,72% out of the total 1,583 tRNA genes present in the nuclear genome were of plastid origin, representing almost 100% of the tRNA genes annotated as plastid. As a result, the total number of tRNA genes encoded by the moringa nuclear genome is significantly higher than the total number of tRNA genes found in other plant genomes, typically ranging between 500 and 600 [67]. As for rRNA genes, we found a significant fraction of complete tRNA genes of plastid origin being functionally expressed. Given the versatility shown by some nuclear tRNAs that are imported and function in the mitochondria [67] or by functional plastid tRNA genes found in the mitochondrial genome [65, 68], it is tempting to speculate at least some plastid tRNAs might also be contributing to the nuclear pool of tRNA involved in cytosolic translation. Indeed, tRNA gene sequences have been shown to evolve rapidly to meet novel translational demands [69]. 27 out of the 112 NUPT nuclear tRNA genes found in the moringa nuclear genome proceeded from tRNA genes annotated as plastid in the plastid genome, which could well indicate the adaptation of their sequences to the new nuclear environment, in a process similar to the concerted evolution of rRNA genes. Two alternative paths for plastid tDNA in the nuclear genome could be to evolve as (i) tRNA-derived RNA fragments (tRFs), or (ii) tRNA-related short interspersed nuclear elements (SINEs). tRFs are a class of sncRNAs identified in all domains of life, a significant fraction of which originate as cleavage products from mature plastid tRNAs and have been attributed to possible regulatory functions within the plant cell as part of signaling pathways [70]. Plastid tRNA genes are also considered a major source of SINEs, a family of small, abundant and highly heterogeneous mobile nonautonomous elements transcribed by RNA PolIII, which rely on the enzymatic machinery of an autonomous long interspersed elements (LINE) partner for propagation by retrotransposition [55, 67, 71]. Although for most families of SINEs, their functions remain unknown and need to be elucidated, there is increasing evidence of their impact on gene function and genome evolution in plants. Similar roles have been hypothesized for RNA genes annotated in the nuclear genome as self-splicing intron, most of which are shown in moringa to be of plastid origin and functionally expressed. The majority of these belonged to group II introns, capable of performing both self-splicing and retrotransposition and also suggested to have a profound impact on nuclear genome evolution. Plastid group II introns likely provided the framework for the emergence of spliceosomal introns and other key components of the spliceosome, eukaryotic retroelements, including telomeres, and other machinery that controls genetic variation and stability [72, 73]. Furthermore, the ability shown by tRNA-derived SINEs and by group II self-splicing introns of plastid origin to experience retrotransposition provides an alternative mechanism to explain their propagation upon arrival in the nuclear genome through repeated duplication.

In addition, a total of 42 *isrR* genes included in the category of regulatory RNA genes were found in the moringa nuclear genome, all of them deriving from a single plastid homolog. *IsrR* genes form a class of specific iron deficiency-responsive antisense RNA genes, whose product binds specifically to the mRNA of the *isiA* gene, which in turn encodes for a protein component of the photosystem, to induce its degradation [74]. Interestingly, *isrR* genes had previously been found only in cyanobacteria [74], although searches in the RFAM database (https://rfam.org/) showed they were present in the nuclear and plastid genomes of other photosynthetic organisms, including plants; in contrast, no homologues of *isiA* genes have been found in plants [75]. As observed for other categories of NUPT RNA genes, many nuclear *isrR* RNA genes are expressed and therefore are likely functional; it remains to be determined what are the actual mRNA targets of nuclear and plastid *isrR* RNA genes in moringa.

In summary, plastid DNA in moringa has a profound impact on the evolution of nuclear genome architecture and function as a major contributor to the nuclear pool of RNA genes, especially those involved in the protein biosynthetic machinery (i.e., rRNA and tRNA genes) and specific subclasses of regulatory RNAs. Furthermore, our results support similar molecular and evolutionary forces would be contributing to the fixation of NUPTs formed in two events separated in time through seemingly disparate mechanisms. An interesting follow-up question is to determine whether these patterns of fixation of NUPTs observed in moringa are species-specific or also apply to other plant species or taxonomic groups.

### Electronic supplementary material

Below is the link to the electronic supplementary material.

Supplementary Material 1

Supplementary Material 2

## Data Availability

No datasets were generated or analysed during the current study.

## Abbreviations

EC: Enzyme Commission
GO: Gene Ontology
KO: KEGG (Kyoto Encyclopedia of Genes and Genomes) Orthology
LINE: Long interspersed element
NUPT: Nuclear plastid DNA sequence
rRF: rRNA-derived RNA fragment
SINE: Short interspersed nuclear element
sncRNA: Small noncoding RNA: RNA sequencing, RNA-seq
TE: Transposable Element
tRF: tRNA-derived RNA fragment
TPM: Transcripts Per Million
