# Supplementary material for: Plastid DNA is a major source of nuclear genome complexity and of RNA genes in the orphan crop moringa
Source: BMC Plant Biol. 2024 May 22;24:437. doi: 10.1186/s12870-024-05158-6 (PMC11110229; doi:10.1186/s12870-024-05158-6)
Supplement: Supplementary file 2 — Supplementary Material 2 [file 12870_2024_5158_MOESM2_ESM.pptx]

## Slide 1
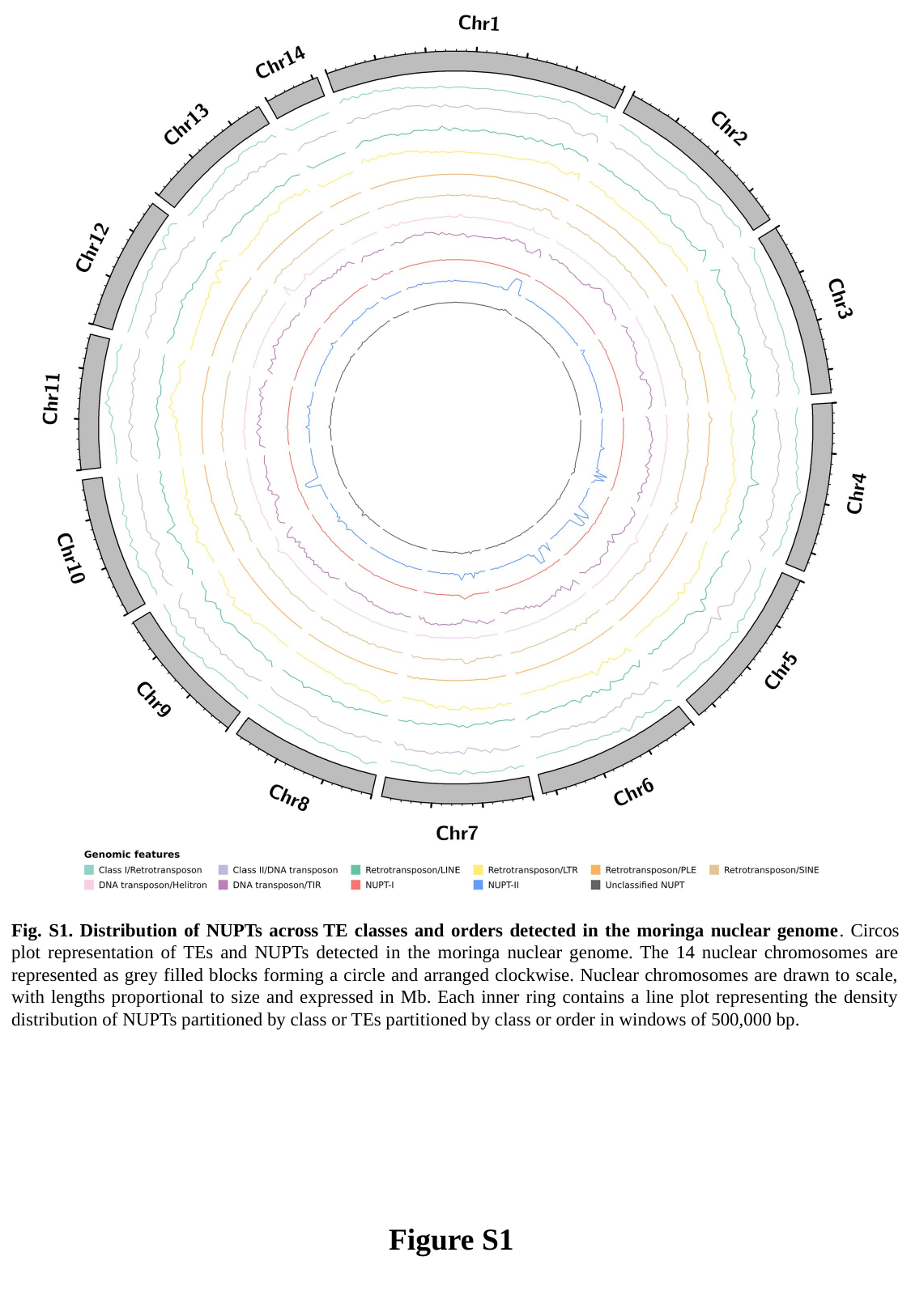

Fig. S1. Distribution of NUPTs across TE classes and orders detected in the moringa nuclear genome. Circos plot representation of TEs and NUPTs detected in the moringa nuclear genome. The 14 nuclear chromosomes are represented as grey filled blocks forming a circle and arranged clockwise. Nuclear chromosomes are drawn to scale, with lengths proportional to size and expressed in Mb. Each inner ring contains a line plot representing the density distribution of NUPTs partitioned by class or TEs partitioned by class or order in windows of 500,000 bp.
Figure S1

## Slide 2
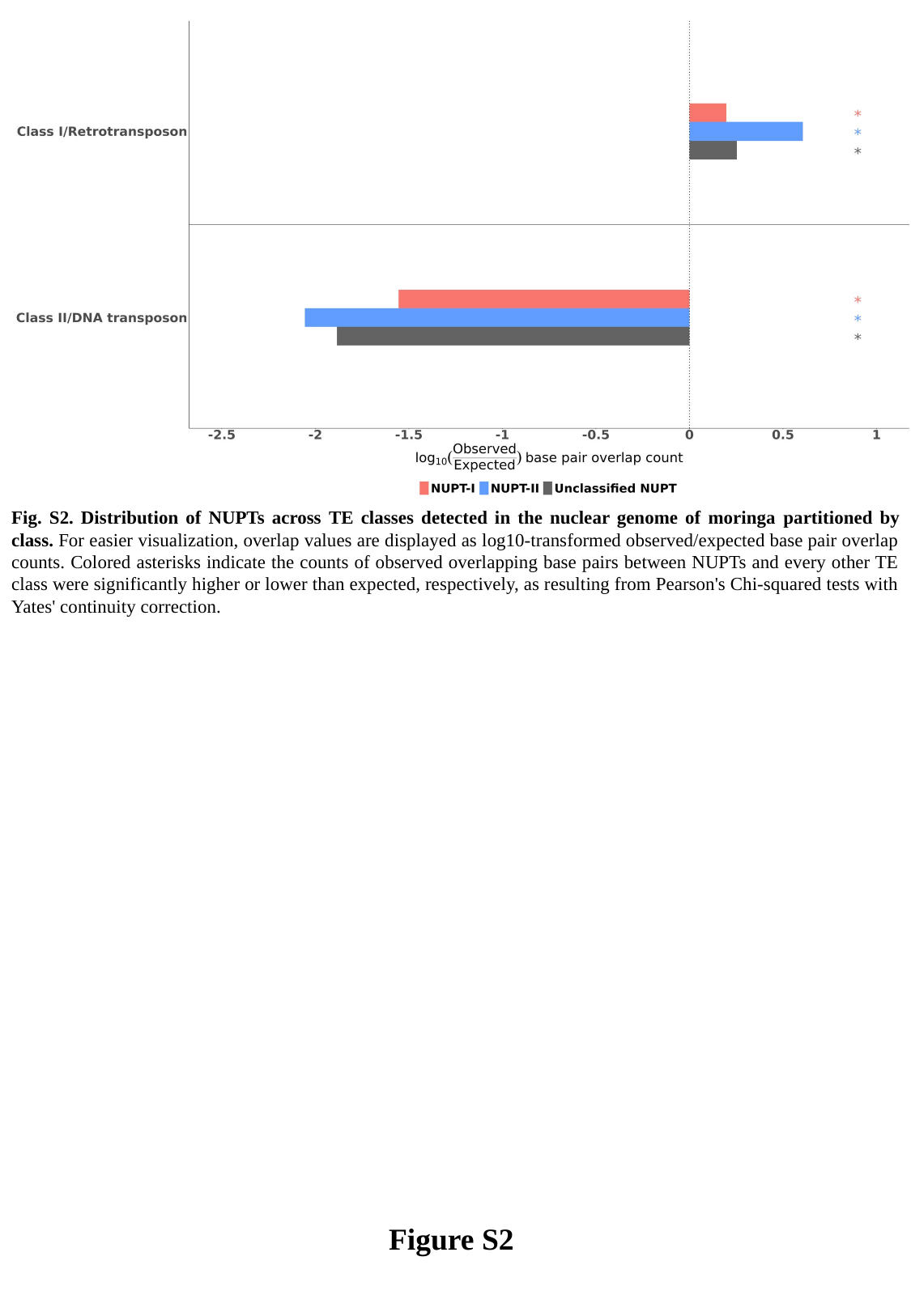

Fig. S2. Distribution of NUPTs across TE classes detected in the nuclear genome of moringa partitioned by class. For easier visualization, overlap values are displayed as log10-transformed observed/expected base pair overlap counts. Colored asterisks indicate the counts of observed overlapping base pairs between NUPTs and every other TE class were significantly higher or lower than expected, respectively, as resulting from Pearson's Chi-squared tests with Yates' continuity correction.
Figure S2

## Slide 3
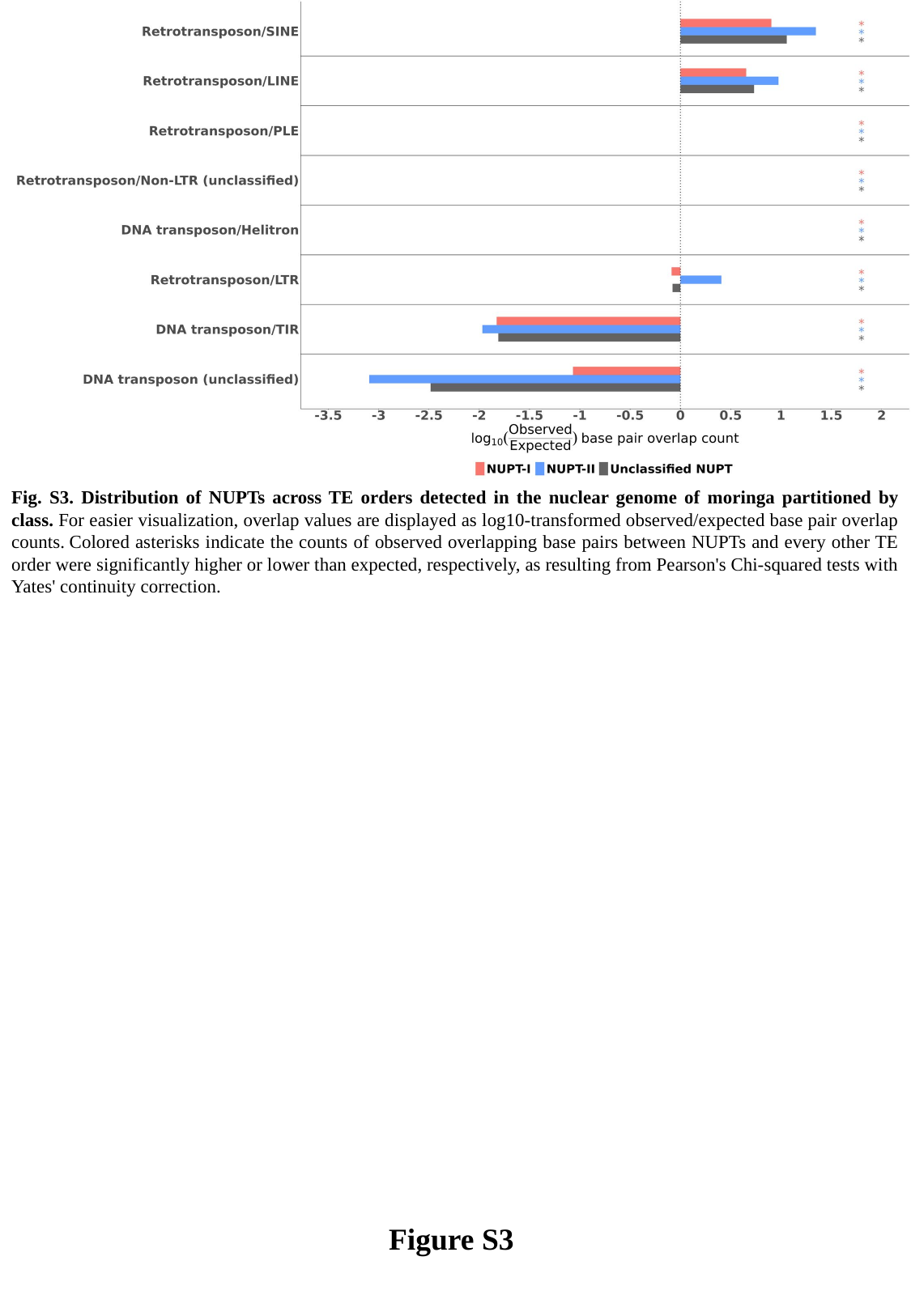

Fig. S3. Distribution of NUPTs across TE orders detected in the nuclear genome of moringa partitioned by class. For easier visualization, overlap values are displayed as log10-transformed observed/expected base pair overlap counts. Colored asterisks indicate the counts of observed overlapping base pairs between NUPTs and every other TE order were significantly higher or lower than expected, respectively, as resulting from Pearson's Chi-squared tests with Yates' continuity correction.
Figure S3

## Slide 4
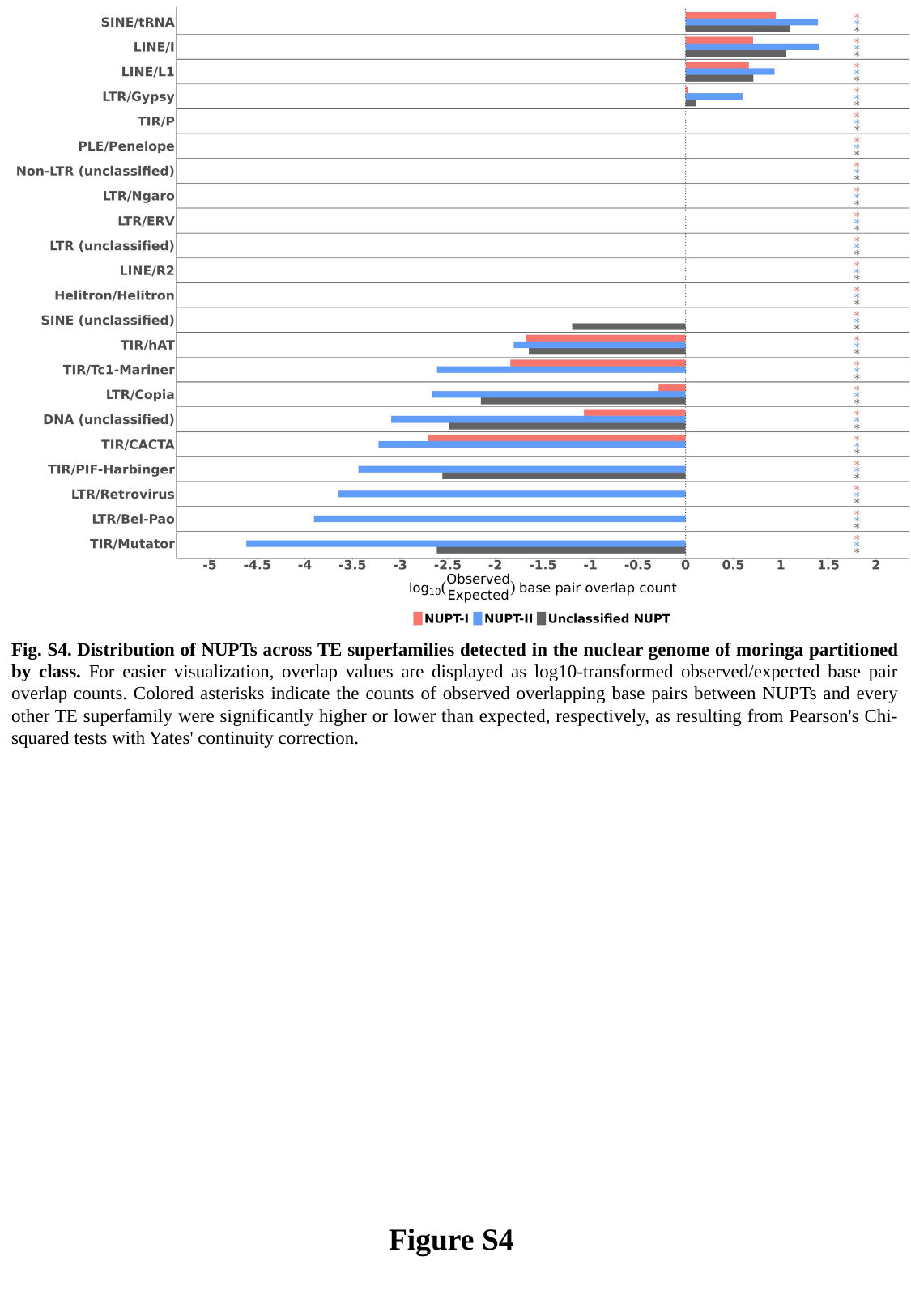

Fig. S4. Distribution of NUPTs across TE superfamilies detected in the nuclear genome of moringa partitioned by class. For easier visualization, overlap values are displayed as log10-transformed observed/expected base pair overlap counts. Colored asterisks indicate the counts of observed overlapping base pairs between NUPTs and every other TE superfamily were significantly higher or lower than expected, respectively, as resulting from Pearson's Chi-squared tests with Yates' continuity correction.
Figure S4

## Slide 5
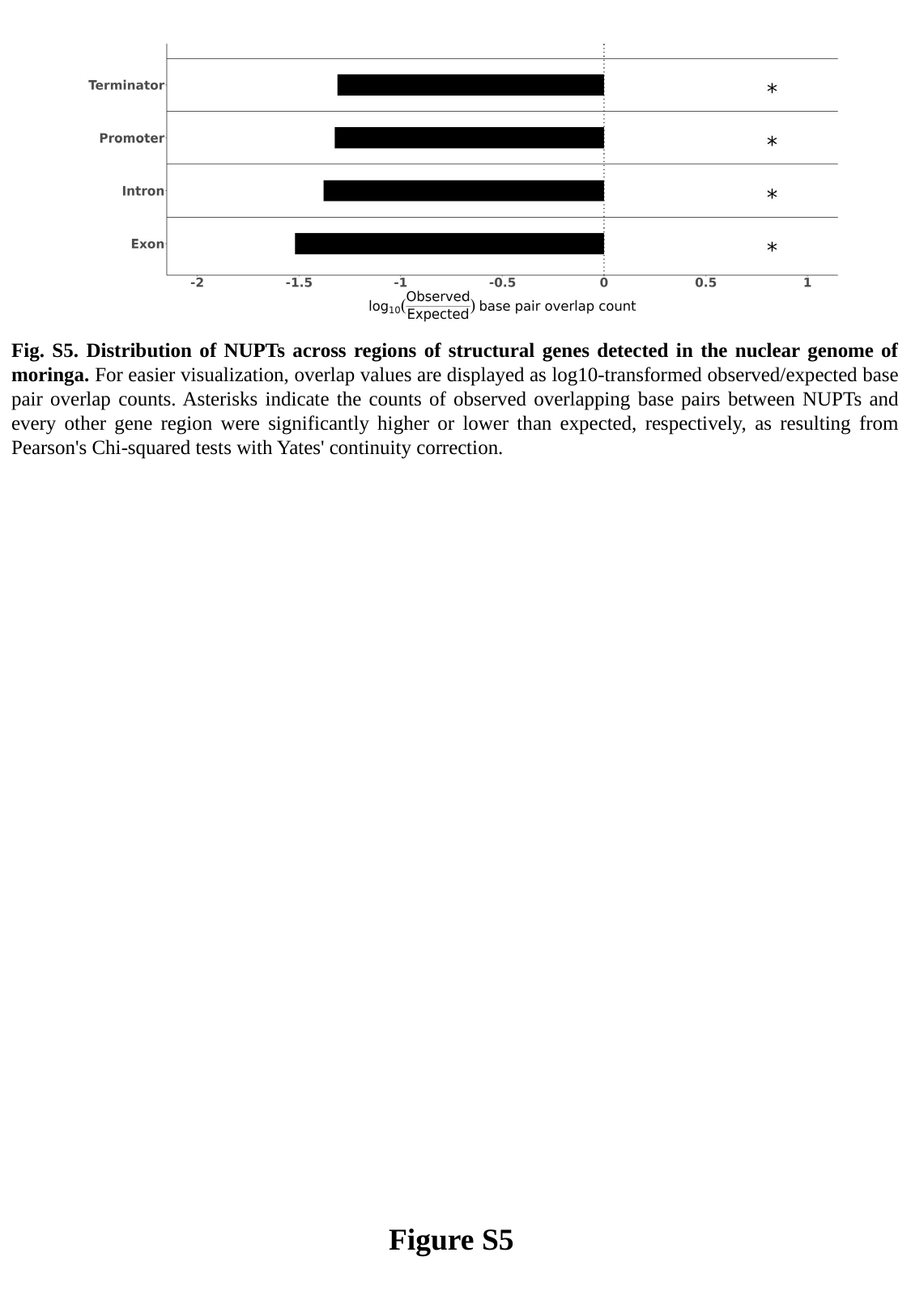

Fig. S5. Distribution of NUPTs across regions of structural genes detected in the nuclear genome of moringa. For easier visualization, overlap values are displayed as log10-transformed observed/expected base pair overlap counts. Asterisks indicate the counts of observed overlapping base pairs between NUPTs and every other gene region were significantly higher or lower than expected, respectively, as resulting from Pearson's Chi-squared tests with Yates' continuity correction.
Figure S5
